# Distinct and Common Large-Scale Networks of the Hippocampal Long Axis in Older Age: Links to Episodic Memory and Dopamine D2 Receptor Availability

**DOI:** 10.1093/cercor/bhab023

**Published:** 2021-03-02

**Authors:** Kristin Nordin, Lars Nyberg, Micael Andersson, Nina Karalija, Katrine Riklund, Lars Bäckman, Alireza Salami

**Affiliations:** Umeå Center for Functional Brain Imaging, Umeå University, S-90187 Umeå, Sweden; Department of Integrative Medical Biology, Umeå University, S-90187 Umeå, Sweden; Wallenberg Centre for Molecular Medicine, Umeå University, S-90187 Umeå, Sweden; Umeå Center for Functional Brain Imaging, Umeå University, S-90187 Umeå, Sweden; Department of Integrative Medical Biology, Umeå University, S-90187 Umeå, Sweden; Wallenberg Centre for Molecular Medicine, Umeå University, S-90187 Umeå, Sweden; Department of Radiation Sciences, Umeå University, S-90187 Umeå, Sweden; Umeå Center for Functional Brain Imaging, Umeå University, S-90187 Umeå, Sweden; Department of Integrative Medical Biology, Umeå University, S-90187 Umeå, Sweden; Umeå Center for Functional Brain Imaging, Umeå University, S-90187 Umeå, Sweden; Department of Radiation Sciences, Umeå University, S-90187 Umeå, Sweden; Umeå Center for Functional Brain Imaging, Umeå University, S-90187 Umeå, Sweden; Department of Radiation Sciences, Umeå University, S-90187 Umeå, Sweden; Aging Research Center, Karolinska Institutet, S-11330 Stockholm, Sweden; Umeå Center for Functional Brain Imaging, Umeå University, S-90187 Umeå, Sweden; Department of Integrative Medical Biology, Umeå University, S-90187 Umeå, Sweden; Wallenberg Centre for Molecular Medicine, Umeå University, S-90187 Umeå, Sweden; Aging Research Center, Karolinska Institutet, S-11330 Stockholm, Sweden

**Keywords:** aging, dopamine, hippocampal axis, PET, resting-state connectivity

## Abstract

The hippocampal longitudinal axis has been linked to dissociated functional networks relevant to episodic memory. However, the organization of axis-dependent networks and their relation to episodic memory in aging remains less explored. Moreover, age-related deterioration of the dopamine (DA) system, affecting memory and functional network properties, might constitute a source of reduced specificity of hippocampal networks in aging. Here, we characterized axis-dependent large-scale hippocampal resting-state networks, their relevance to episodic memory, and links to DA in older individuals (*n* = 170, 64–68 years). Partial least squares identified 2 dissociated networks differentially connected to the anterior and posterior hippocampus. These overlapped with anterior–temporal/posterior–medial networks in young adults, indicating preserved organization of axis-dependent connectivity in old age. However, axis-specific networks were overall unrelated to memory and hippocampal DA D2 receptor availability (D2DR) measured with [^11^C]-raclopride positron emission tomography. Further analyses identified a memory-related network modulated by hippocampal D2DR, equally connected to anterior–posterior regions. This network included medial frontal, posterior parietal, and striatal areas. The results add to the current understanding of large-scale hippocampal connectivity in aging, demonstrating axis-dependent connectivity with dissociated anterior and posterior networks, as well as a primary role in episodic memory of connectivity shared by regions along the hippocampalaxis.

## Introduction

Evidence indicates that the organization of cortical input to the hippocampus is heterogeneous along its longitudinal axis, shaping the role of anterior and posterior hippocampal regions in declarative memory (Ranganath and Ritchey 2012; Poppenk et al. 2013; Strange et al. 2014; Grady 2019). In younger adults, assessments of functional connectivity measured during rest have demonstrated a long-axis gradient of connectivity both within the hippocampus (Brunec et al. 2018; vos de Wael et al. 2018; Dalton et al. 2019b), and in its connectivity with large-scale networks (Kahn et al. 2008; Robinson et al. 2016; Plachti et al. 2019; Przeździk et al. 2019; Zhong et al. 2019). There are, however, few studies assessing the organization and functional relevance of anterior and posterior hippocampal connectivity at older age—when decline in both episodic memory and its hippocampal correlates is commonly observed (Rönnlund et al. 2005; Josefsson et al. 2012; Bettio et al. 2017; Gorbach et al. 2017; Nyberg 2017).

Largely corresponding to anatomical connections observed in animal studies (Suzuki and Amaral 1994; Burwell 2006; Aggleton 2012), resting-state studies have shown distinct cortical connectivity of the anterior and posterior hippocampus by way of their respective links to the perirhinal and parahippocampal cortices (Libby et al. 2012; Maass et al. 2015; Kaboodvand et al. 2018; Dalton et al. 2019b). Primary cross-talk is commonly reported between the anterior hippocampus and anterior temporal and ventromedial prefrontal areas, whereas the posterior hippocampus is more functionally connected to medial frontal and posterior parietal areas (Kahn et al. 2008; Qin et al. 2016; Robinson et al. 2016; Wagner et al. 2016). Although this type of axis-specific connectivity has been linked to episodic memory in younger adults (Poppenk and Moscovitch 2011; Adnan et al. 2016; Persson et al. 2018), there are several examples of hippocampal connectivity deviating from this type of anterior–temporal/posterior–medial framework (Ranganath and Ritchey 2012). Alternative accounts are mainly characterized by predominant links between the anterior hippocampus and regions of the default mode network (DMN; Raichle et al. 2001; Buckner et al. 2008), including posterior–medial areas such as the posterior cingulate cortex (PCC), retrosplenial cortex, and the precuneus (Vincent et al. 2006; Chase et al. 2015; Blessing et al. 2016; Kaboodvand et al. 2018; Zhong et al. 2019). Adding to the overall discrepancy among findings, some studies find greater overlap than separation of anterior and posterior networks (Blessing et al. 2016; Wang et al. 2016).

Importantly, age-related variations in the organization of axis-specific networks have been less explored. One study demonstrated an anterior–temporal/posterior–medial division in resting-state connectivity in older adults, suggesting that the organization of such axis-specific networks might be relatively preserved in normal aging (Das et al. 2015). On the other hand, it is conceivable that processes related to aging affect certain aspects of network organization and networks’ associations with episodic memory. For instance, longitudinal age-related increases in connectivity within the posterior medial temporal lobe (MTL), accompanied by a decoupling of these areas from the DMN, have been demonstrated to have negative consequences for episodic memory (Salami et al. 2016). Moreover, this study showed decreased connectivity over time within the anterior MTL. Cross-sectional studies have observed similar axis-dependent effects of age, both within the hippocampus/MTL (Dalton et al. 2019a; Stark et al. 2019), as well as in relation to the DMN (Damoiseaux et al. 2016).

Large-scale resting-state networks, in general, become less segregated in older age, possibly reflecting a loss of specificity in neuronal signal accompanied by dedifferentiation among networks (Betzel et al. 2014; Chan et al. 2014; Geerligs et al. 2015; Damoiseaux 2017; Wig 2017). Given that dopamine (DA) exerts an enhancing effect on specificity in neuronal signal through modulation of synaptic activity (Seamans and Yang 2004; Shafiei et al. 2019; Shine et al. 2019), the age-sensitivity of the DA system might constitute one source of reduced distinctiveness of hippocampal large-scale networks in aging. Various DA markers have been found to be reduced in older, compared to younger adults, including the DA D2 receptors (D2DRs) (Rinne et al. 1993; Volkow et al. 1996; Kaasinen et al. 2000; Karrer et al. 2017; Seaman et al. 2019). Moreover, both hippocampal and striatal D2DR status has been linked to episodic memory function (Bäckman et al. 2000, 2010; Kaasinen and Rinne 2002; Nyberg et al. 2016). Nyberg et al. (2016) further showed increased functional connectivity between the caudate nucleus and the MTL as a function of caudate D2DR. Yet, the extent to which hippocampal D2DR modulates axis-specific hippocampal networks and their association with episodic memory in older age remains unknown.

Given these remaining questions regarding hippocampal connectivity in aging, the main aim of this study was to assess the extent and organization of axis-specific large-scale hippocampal networks in older adults, their relevance to episodic memory performance, and potential modulation by D2DR. In addition, we also sought to examine associations between hippocampal networks and measures of structural and vascular hippocampal integrity. Aging is associated with loss of hippocampal gray matter (GM) (Raz et al. 2005; Fjell et al. 2009), linked to longitudinal episodic memory decline (Kramer et al. 2007; Gorbach et al. 2017). Importantly, age-related atrophy might vary along the hippocampal axis (Malykhin et al. 2017; Langnes et al. 2020). Effects are often reported as most pronounced in the anterior hippocampus across older samples (≥ 50 years) (Jack et al. 1997; Hackert et al. 2002; Chen et al. 2010; Nordin et al. 2017), whereas atrophy in the posterior hippocampus has been reported as most pronounced across samples including both younger and older adults (Malykhin et al. 2008; Kalpouzos et al. 2009; Nordin et al. 2018). Furthermore, perfusion of the hippocampus and surrounding MTL structures has been positively linked to episodic memory performance and task-related activation in older adults, suggesting a potential role of perfusion also to memory-related hippocampal functional networks (Bangen et al. 2009).

In this study, we characterized large-scale resting-state networks of regions along the hippocampal axis in a large, age-homogeneous, sample of healthy older adults (*n* = 170, 64–68 years), using multivariate partial least squares (PLS). We hypothesized that participants with high levels of hippocampal D2DR and episodic memory would display high expression of distinct axis-dependent hippocampal networks, reflecting preserved network organization and specificity in aging (Nyberg et al. 2012; Zuo et al. 2020).

## Materials and Methods

The analyses were performed with data from the Cognition, Brain, and Aging (COBRA) study for which the design, imaging protocols, cognitive testing, and lifestyle assessments have been described elsewhere (Nevalainen et al. 2015; Nyberg et al. 2016; Köhncke et al. 2018; Salami et al. 2019). Here we describe the materials and methods directly relevant to the current study. The COBRA study was approved by the Regional Ethical board and the Local Radiation Safety Committee of Umeå, Sweden. All participants provided written informed consent prior to testing.

### Participants

The original COBRA sample included 181 participants (64–68 years; mean age = 66.2, ± 1.2; 100 men and 81 women) recruited by postal mail to a random sample drawn from the population register of Umeå in northern Sweden. Individuals with suspected brain pathology, impaired cognitive functioning (Mini Mental State Examination < 27), and conditions that could bias measurements of brain and cognition (e.g., diabetes, tumors, and trauma) or hinder imaging sessions (e.g., metal implants) were ineligible to participate. Eleven participants were excluded from the original sample due to technical issues such as magnetic resonance–positron emission tomography (MR-PET) image coregistration and excessive in-scanner movement (frame-wise displacement [FD] >0.5; Power, 2012). Thus, the final number of participants included in the present study was 170 (64–68 years; mean age = 66.9, ± 1.2; 92 men and 78 women).

### Episodic Memory

Three tasks including verbal, numerical, and figural material, respectively, were used to assess episodic memory. These tested word recall, number–word recall, and object–location recall (Nevalainen et al. 2015). In the word recall task, participants were presented with 16 Swedish concrete nouns that appeared consecutively on a computer screen. Each word was presented for 6 s during encoding with an interstimulus interval (ISI) of 1 s. Following list presentation, participants reported as many words as they could recall by typing them on the keyboard. Two trials were completed, yielding a maximum score of 32.

In the number–word task, participants memorized pairs of 2-digit numbers and concrete plural nouns (e.g., 46 dogs). Eight number–word pairs were presented during encoding, each displayed for 6 s, with an ISI of 1 s. Following encoding nouns were presented again, now in a different order than during encoding, and participants had to report the 2-digit number associated with each presented noun (e.g., How many dogs?). This task was administered in 2 trials with a total maximum score of 16.

The third task was an object–location memory task. Here, participants were presented with a 6 × 6 square grid in which 12 objects were, one by one, shown at distinct locations. Each object-position pairing was displayed for 8 s, with an ISI of 1 s. Following encoding, all objects were simultaneously shown next to the grid, ready to be moved by the participant (in any order) to its correct position in the grid. If unable to recall the correct position of an object, participants had to make a guess and place the object somewhere in the grid to the best of their ability. Two trials of this task were administered, making the total maximum score 24.

A composite score of performances across these 3 tasks was calculated and used as the measure of episodic memory. For each of the 3 tasks, scores were summarized across the total number of trials. The 3 resulting sum scores were *z*-standardized and averaged to form 1 composite episodic-memory score (*T* score: mean = 50; standard deviation [SD] = 10). Finally, missing values (<1.2% for all variables) were replaced by the average of the available observed scores.

### Image Acquisition

Brain imaging was conducted at Umeå University Hospital, Sweden. Structural and functional MRI data were acquired with a 3 Tesla Discovery MR 750 scanner (General Electric), using a 32-channel head coil. PET data were acquired with a Discovery PET/computed tomography (CT) 690 scanner (General Electric).

#### Structural MRI

Anatomical *T*_1_-weighted images were acquired with a 3D fast-spoiled echo sequence, collected as 176 slices with a thickness of 1 mm. Original voxel size was 0.98 × 0.98 × 1.0 mm, reconstructed to 0.49 × 0.49 × 1.0 mm. TR = 8.2 ms, flip angle = 12 degrees, field of view = 25 × 25 cm.

#### Arterial Spin Labeling (ASL)

Perfusion data were acquired using 3D pseudo-continuous ASL with background suppression and spiral read-out. Labeling time was 1.5 s and postlabeling delay time 1.5 s. Field of view was 24 cm, with a slice thickness of 4 mm, and the acquisition resolution was 8 × 512 (arms × data points), with the number of averages set at 3. Spatial resolution was reconstructed to 1.88 × 1.88 × 4.0 mm. This yielded whole-brain perfusion in mL/100 g/min. Estimation of hippocampal perfusion was made using anterior, middle, and posterior hippocampal regions of interest (ROIs), which were based on resting-state parcellation of the hippocampus, described furtherdown.

#### Functional MR Imaging

Functional MR time series were collected during a resting-state condition (5:40 min), for which participants were instructed to keep their eyes open and look at a fixation cross. A *T*_2_*-weighted single-shot gradient echoplanar-imaging sequence with the following parameters was used to collect a total of 170 volumes: 37 transaxial slices, 3.4 mm thickness, 0.5 mm spacing, voxel size 2.60 × 2.60 × 3.9 mm, reconstructed to 1.95 × 1.95 × 3.9 mm, TE/TR = 30/2000 ms, 80 degrees flip angle, 25 × 25 cm field of view, and a 96 × 96 acquisition matrix (Y direction phase encoding). Ten dummy scans were collected at the start of the sequence.

#### PET Imaging

PET data were acquired during a resting-state condition following an intravenous bolus injection of 250 MBq [^11^C]-raclopride. A low-dose helical CT scan (20 mA, 120 kV, 0.8 s/revolution) was obtained prior to the injection, for the purpose of attenuation correction. After the bolus injection, a 55 min 18-frame dynamic scan was acquired. Attenuation- and decay-corrected images (47 slices, field of view = 25 cm, 256 × 256-pixel transaxial images, voxel size = 0.977 × 0.977 × 3.27 mm) were reconstructed with the iterative algorithm VUE PointHD-SharpIR (GE; Bettinardi et al. 2011), using 6 iterations, 24 subsets, and 3.0-mm postfiltering. An individually fixed thermoplastic mask attached to the PET scanner bed surface was used to minimize head movements during image acquisition. The average time between magnetic resonance imaging (MRI) and PET sessions were 3.9 ± 5.7 days, with PET conducted 2 days after the MR session for 82% of the individuals.

### Image Preprocessing

#### Structural MR Images

For delineation of the hippocampus, a mean image of normalized individual *T*_1_-weighted images was submitted to automated segmentation in FreeSurfer version 6 (Fischl et al. 2002, 2004). This yielded sample-specific segmentations of the left and right hippocampus, in a later step used for definition of anterior, middle, and posterior hippocampal ROIs using functional images. To estimate GM volume for each participant, the resulting axis ROIs were denormalized to native space, first by an affine transformation to dartel-template space and then applying the flow field files for each subject. Volume estimates were subsequently corrected for total intracranial volume (ICV) using a covariance approach, in which adjusted hippocampal volume = raw hippocampal volume−*b*(ICV−mean ICV), where *b* is the regression slope of the hippocampal ROI volume on ICV (Jack et al. 1989; Free et al. 1995; Buckner et al. 2004). Covariance adjustment has been suggested as more efficient in removing effects of ICV, compared with proportion adjustment, which risk introducing systematic error due to the lack of proportionality between regional volumes and ICV (Voevodskaya et al. 2014; Pintzka et al. 2015).

#### Resting-State Functional Magnetic Resonance Imaging (fMRI)

The fMRI data were preprocessed using the Data Processing Assistant for Resting-State fMRI (DPARSF version 4.4; Yan and Zang 2010, http://rfmri.org/DPARSF) software, and the Data Processing & Analysis of Brain Imaging (DPABI version 3.1; Yan et al. 2016, http://rfmri.org/DPABI) toolbox implemented in Statistical Parametric Mapping 12 (SPM12: www.fil.ion.ucl.ac.uk/spm). The data were slice-timing corrected, motion corrected, and within-subject rigid registration was done to align functional images with anatomical *T*_1_ images. Mean FD across the sample was 0.19 mm (SD = 0.09, range = 0.05–0.45 mm). Physiological noise was removed by regressing out Friston’s 24 parameters (Yan et al. 2013) and nuisance variables such as white matter and cerebrospinal fluid signal. Data were then high pass filtered (*f* > 0.008 Hz) before finally normalized into MNI space with a spatial resolution of 1.5 × 1.5 × 1.5 mm^3^, by use of a sample-specific template created by Diffeomorphic Anatomical Registration Through Exponentiated Lie Algebra (DARTEL; Ashburner 2007), and then smoothed using a 6.0-mm full width at half maximum Gaussian filter.

### Functional Parcellation of the Hippocampus

For definition of anterior, middle, and posterior hippocampal ROIs, temporal concatenation independent-component analysis (ICA), implemented in the GIFT toolbox (Calhoun et al. 2001a, 2001b; Allen et al. 2011), was conducted on the preprocessed resting-state data and restricted to the hippocampus by use of the sample-specific left and right hippocampal FreeSurfer segmentations. This data-driven approach enables the identification of spatially independent, but temporally coherent, components of brain data and has been successful in delineating regions along the hippocampal/MTL axis (Blessing et al. 2016; Salami et al. 2016). The details of ICA analyses have been provided in our earlier work (Salami et al. 2014, 2016).

**Figure 1:**
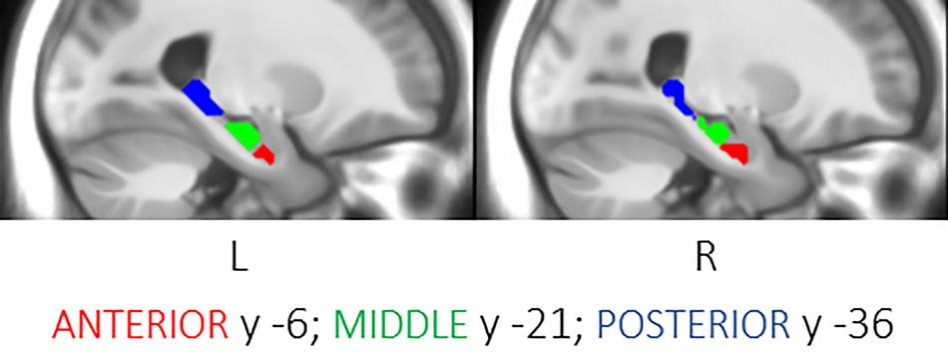
Hippocampal ROIs from resting-state based parcellation of the anterior (red), middle (green) and posterior (blue) hippocampus through independent component analysis.

In total, 19 components in the left hemisphere and 18 components in the right hemisphere were identified. These were spread out along the hippocampus’ longitudinal axis from MNI coordinates *y* − 6 to −36 in both hemispheres. Five components in both hemispheres were identified on the same MNI coordinates. One was located in the anterior hippocampus (aHC) with its center at *y* = −6; 2 were located in the middle hippocampus (mHC) at *y* = −18 and −21, and 2 were located in the posterior hippocampus (pHC) at *y* = −33 and −36. These 5 components were selected based on their correspondence between hemispheres and their contribution to ROIs covering the greatest extension of the hippocampal axis. The 2 middle components and the 2 posterior components were combined into 1 middle ROI and 1 posterior ROI, respectively (Fig. 1). Right and left ROIs were combined into bilateral ROIs representing the aHC (82 voxels), the mHC (283 voxels), and the pHC (157 voxels). To control for potential effects of differences in signal-to-noise ratios (SNR) between hippocampal ROIs, we estimated the SNR for each ROI by averaging over the ROI and dividing the mean signal for that region with its SD and used the resulting estimates as covariates in subsequent analyses. SNR increased in an anterior-to-posterior manner (aHC: M = 292.8, SD = 107.1; mHC: M = 428.6, SD = 126.4; pHC M = 590.3, SD = 159.6), with differences between regions observed as statistically significant (aHC < mHC: *t*_166_ = −16.0, *P* < 0.001); mHC < pHC: *t*_166_ = −17.2, *P* < 0.001; aHC < pHC: *t*_166_ = −28.2, *P* < 0.001). Whole-brain correlation maps, in which each voxel comprises the correlation value between the time series of that voxel and a specified seed region, were calculated and *z*-transformed in DPARSF, for each of the anterior, middle, and posterior hippocampal ROIs, separately. These correlation maps were later used as input to PLS analyses of axis-dependent functional connectivity. A visualization of each hippocampal region’s correlation map (i.e., whole-brain connectivity) is presented in Supplementary Materials.

### Analyses of Hippocampal Resting-State Connectivity

Hippocampal resting-state connectivity was assessed by submitting aHC, mHC, and pHC correlation maps into PLS analyses (McIntosh et al. 1996; McIntosh and Lobaugh 2004; Krishnan et al. 2011). PLS is a data-driven multivariate method that identifies spatial patterns in the brain expressing the optimal association between brain voxels and experimental condition (in this case hippocampal regions). The method acts on the across-subject correlation matrix between voxel values and the specified design/conditions and identifies orthogonal latent variables (LVs) through singular-value decomposition. Every brain voxel has a weight on each LV, a salience that can be either positive or negative, corresponding to its covariance with the pattern captured by the LV. These saliences are used to derive brain scores, indicating how strongly an LV is expressed in each participant’s brain image. For each participant, brain scores are calculated by multiplying the value in each voxel by the salience of that voxel and finally summing across all voxels.

Two separate analyses, corresponding to 2 separate research questions, were conducted. The first PLS, seeking to maximally explain predefined variances (i.e., aHC, mHC, and pHC connectivity) was used to assess connectivity across the 3 hippocampal regions independent of any other measure. A second, behavioral PLS, was used to identify connectivity optimally explaining individual differences in episodic memory performance and hippocampal D2DR. Correlation maps belonging to the aHC, mHC, and pHC were entered into PLS using its structural module, which operates on 1 image per participant per condition, in contrast to fMRI modules operating on several images across time (for a review see Krishnan et al. 2011). Memory and D2DR were entered as covariates in the second analysis. The cross-block correlation matrix was computed between connectivity of regions along the hippocampal axis and episodic memory/D2DR, suchthat.

*R* = *Y*^T^*X*.

where *X* was correlation maps and *Y* was our episodic memory and hippocampal D2DR variables.

Significance of identified LVs was in this study determined by 1000 permutations, and the reliability of each voxel’s contribution to an LV was assessed by 1000 bootstraps, estimating salience’s standard errors. Voxels with a ratio between salience and standard error (bootstrap ratio [BSR]) of ≥3.3 (which corresponds to *P* < 0.001) were considered making reliable contributions to the LVs. All reliable clusters comprised contiguous voxels (minimum cluster sixe 10 voxels), with a distance between clusters of at least 10 mm. Moreover, the upper and lower percentiles of the bootstrap distribution were used to generate 95% confidence intervals (CIs) around the brain scores to facilitate interpretation. For instance, a significant difference between brain scores pertaining to different hippocampal regions is indicated by nonoverlapping CIs. Similarly, brain or correlation scores were considered unreliable when CIs overlapped withzero.

#### PET Images

Preprocessing of PET images was performed in SPM8 (www.fil.ion.ucl.ac.uk/spm). The 18-frame PET scans were first coregistered to the anatomical *T*_1_ image with the time frame mean of PET images used as source. For determination of D2DR binding potential, the ratio of specifically bound radioligand to nondisplaceable radioligand in tissue (Innis et al. 2007), time–activity curves for the left and right hippocampus, defined by the segmentation performed in FreeSurfer, were entered into Logan analyses (Logan et al. 1990). As reference, time–activity curves in the GM of cerebellum were used. Left and right hippocampal values were averaged into a bilateral hippocampal D2DR measure. Given known limitations in reliably assessing extrastriatal D2DR due to its relatively low densities outside striatal regions, we chose to use a whole-hippocampus measure rather than separate measures for our aHC, mHC, and pHCROIs.

## Results

### Episodic Memory

Participants performed at similar levels on all 3 subtests of episodic memory: word recall *M* = 49.98 ± 9.98; number–word recall *M* = 49.99 ± 9.83; object–location recall M = 49.85 ± 9.66, with the composite episodic memory score showing a mean of 49.87 ± 7.51. For an overview of the factor structure of the episodic memory tests, assessed through structural equation modeling, see Nevalainen et al. 2015.

### Dissociated Anterior and Posterior Resting-State Networks

**Figure 2:**
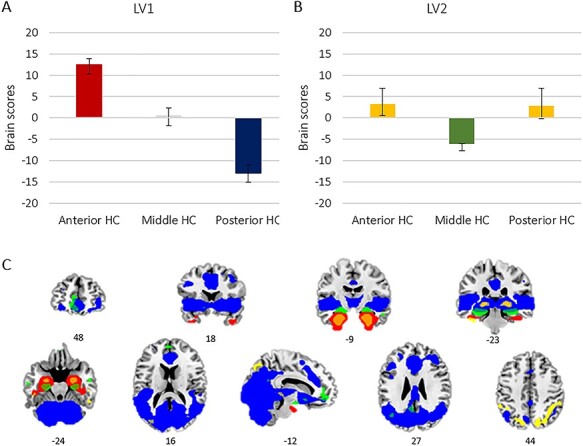
Hippocampal networks identified by PLS. (*A*) Dissociation of anterior and posterior hippocampal connectivity. Bars (with 95% CI) represent mean brain scores pertaining to connectivity of each hippocampal region, expressing associations between connectivity of each region and the pattern exhibited by LV1. (*B*) Dissociation of middle hippocampal connectivity from connectivity shared by the anterior and posterior hippocampus. Bars (with 95% CI) represent mean brain scores pertaining to connectivity of each hippocampal region, expressing associations between each region’s connectivity and the pattern exhibited by LV2. (*C*) Brain regions primarily connected to the anterior hippocampus (red); to the posterior hippocampus (blue); connectivity shared by the anterior and posterior hippocampus (yellow); regions primarily connected to the middle hippocampus (green).

Correlation maps for the anterior, middle, and posterior hippocampal ROIs were entered as separate conditions into a PLS analysis assessing the degree to which large-scale connectivity varied as a function of the hippocampal axis. The primary LV identified by the PLS accounted for 85% of the variance (*P* < 0.001) and exhibited a spatial pattern dissociating connectivity of the aHC and pHC, whereas the contribution of mHC connectivity to the identified pattern was unreliable (CIs crosses zero; Fig. 2). The lack of clear contribution of mHC connectivity to either the anterior or the posterior network suggests that both networks might be equally represented within connectivity of the mHC. More specifically, clear correspondence of mHC connectivity to either the anterior or posterior network shows interindividual variation such that its anterior/posterior characteristics are attenuated on a group level.

Connectivity primarily linked to the aHC extended to a network including temporal and frontal regions, such as the perirhinal cortex, the temporal pole, the inferior temporal gyrus, the inferior orbitofrontal gyrus, and the fusiform gyrus. In contrast, connectivity primarily linked to the pHC constituted a larger network extending to parietal, medial, occipital, and striatal regions. This network included the fusiform gyrus, thalamus, caudate nucleus, regions in the cingulate cortex, middle orbitofrontal gyrus, superior and middle frontal gyrus, angular gyrus, precuneus, lingual gyrus, middle occipital cortex, and cerebellum (Table 1).

**Table 1:** Regions in the anterior and posterior hippocampal networks

	*x*, *y*, *z*	Cluster size	BSR
Anterior hippocampal network			
Parahippocampal gyrus, R	24, −9, −24	1058	36.1
Fusiform gyrus, R	42, −33, −27		5.3
Parahippocampal gyrus, L	−24, −9, −27	1047	31.5
Middle temporal pole, L	−33, 15, −42		7.7
Superior temporal pole, L	−21, 12, −30		6.0
Inferior temporal gyrus, L	−48, −21, −27		4.7
Cerebellum, L	−36, −36, −27		4.5
Inferior orbitofrontal gyrus, R	21, 18, −27	10	4.2
			
Posterior hippocampal network			
Hippocampus, L	−24, −36, −3	28 943	31.3
Hippocampus, R	24, −36, 0		29.2
Lingual gyrus, L	−6, −66, 6		10.8
Lingual gyrus, R	9, −63, 0		10.1
Calcarine fissure, L	21, −60, 6		10.3
Calcarine fissure, R	9, −75, 12		10.4
Thalamus, L	−9, −15, 3		10.3
Thalamus, R	9, −15, 3		10.0
Insula, L	−30, 15, −9		9.8
Insula, R	39, 3, 12		4.1
Cerebellum, L	−27, −72, −33		9.8
Cerebellum, R	33, −66, −33		9.7
Middle occipital gyrus, L	−39, −60, 0		9.5
Middle occipital gyrus, R	36, −66, 36		5.8
Olfactory cortex, L	−15, 12, −12		9.3
Putamen, R	24, 21, −6		9.1
Middle temporal gyrus, L	−51, −15, −9		7.8
Middle temporal gyrus, R	54, −57, 9		6.0
Anterior cingulate cortex, L	−3, 33, 27		7.1
Anterior cingulate cortex, R	6, 27, 30		7.7
Fusiform gyrus, L	−30, −72, −12		7.3
Fusiform gyrus, R	30, −75, −6		7.6
Superior temporal gyrus, L	−51, −27, 9		7.3
Caudate nucleus, R	9, 9, −3		7.3
Cuneus, R	12, −78, 33		7.2
Heschl’s gyrus, L	−36, −21, 6		6.9
Middle cingulate cortex, L	−6, 21, 33		6.8
Middle cingulate cortex, R	3, −36, 39		6.5
Precuneus, L	−6, −72, 42		6.6
Middle orbitofrontal gyrus, L	−30, 48, −12		5.2
Middle orbitofrontal gyrus, R	3, 48, −9		6.5
Angular gyrus, L	−45, −63, 39		6.5
Angular gyrus, R	42, −48, 36		6.2
PCC, L	−6, −39, 33		6.1
Supramarginal gyrus, R	51, −39, 39		5.7
Middle frontal gyrus, L	−42, 24, 33		5.0
Middle frontal gyrus, R	39, 48, 0		5.4
Superior orbitofrontal gyrus, L	−27, 57, 0		4.4
Superior frontal gyrus, L	−24, 33, 33		4.3
Superior frontal gyrus, R	18, 15, 57		4.1
Precentral gyrus, L	−45, 3, 39		4.0
Supplemental motor area, L	−6, 3, 69		3.9
Inferior frontal gyrus, R	51, 12, 15		3.7

Note: Regions presented indented and without specified cluster size are subsidiary maxima of main clusters. Minimum cluster size = 10 voxels.

A second significant LV accounting for 15% of the variance (*P* < 0.01) identified a network similarly associated with the anterior and posterior hippocampus, distinct from connectivity of the mHC, although the association of pHC connectivity with this shared network was found to be unreliable (CIs crosses zero; Fig. 2). In contrast to the mHC, which showed significant connectivity with the olfactory cortex, middle temporal gyrus, caudate, middle orbitofrontal gyrus, precuneus, and angular gyrus, the network linked to both the aHC and pHC comprised the putamen, thalamus, the inferior temporal gyrus, the superior and inferior parietal cortex, and cuneus (Table 2).

**Table 2:** Regions in the network shared by the anterior and posterior hippocampus; regions in the network specific to the middle hippocampus

	*x*, *y*, *z*	Cluster size	BSR
Anterior and posterior hippocampal network			
Parahippocampal gyrus, L	−24, −9, −27	222	14.2
Putamen, L	−30, −3, −48		4.3
Inferior temporal gyrus, L	−48, −30, −27		4.1
Cuneus, R	15, −78, 45	220	4.3
Inferior parietal cortex, R	42, −45, 39		4.1
Superior occipital gyrus, R	27, −72, 45		3.8
Supramarginal gyrus, R	54, −36, 39		3.6
Parahippocampal gyrus, R	24, −9, −27	216	16.5
Hippocampus, R	24, −36, 0	173	10.3
Thalamus, R	15, −27, 12		3.6
Hippocampus, L	−24, −36, 0	166	12.1
Superior parietal cortex, L	−12, −78, 48	74	4.3
Precuneus, L	−9, −69, 54		3.6
Inferior parietal cortex, L	−39, −48, 42	28	3.8
Inferior temporal gyrus, L	57, −42, −27	25	4.0
			
Middle hippocampal network			
Hippocampus, L	−24, −18, −18	367	23.7
Parahippocampal gyrus, R	27, −18, −18	300	23.5
Cerebellum, R	15, −33, −15		4.3
Middle orbitofrontal gyrus, L	−9, 51, −9	211	4.3
Anterior cingulate cortex, L	−9, 48, 9		4.0
Rectus, L	−9, 39, −18		3.8
Rectus, R	3, 48, −21		3.8
Medial superior frontal gyrus, L	−3, 57, 15		3.8
Precuneus, L	−9, −54, 24	43	4.4
Calcarine fissure, L	−6, −63, 12		3.6
Angular gyrus, L	−42, −66, 27	34	4.0
Olfactory cortex, R	12, 12, −12	24	4.4
Middle temporal gyrus, L	−60, −12, −21	19	3.7
Middle temporal gyrus, R	54, −9, −21	10	4.1
Caudate nucleus, L	−9, 12, −9	16	4.2

Note: Regions presented indented and without specified cluster size are subsidiary maxima of main clusters. Minimum cluster size = 10 voxels.

Mean brain scores showed the same pattern across anterior, middle, and posterior hippocampal regions after regressing out SNR, for both LV1 (aHC: 13.24 vs. 17.50; mHC: 1.25 vs. 1.51; pHC: −12.21 vs. −16.78) and LV2 (aHC: 3.16 vs. 3.0; mHC: −6.34 vs. −6.33; pHC: 2.74 vs. 2.99).

#### Axis-Dependent Connectivity in Relation to Episodic Memory

PLS brain scores indicate to what degree connectivity of a participants’ aHC, mHC, and pHC contributes to the identified networks of an LV (i.e., to what degree LV1 and LV2 networks are expressed in connectivity of a participant’s aHC, mHC, and pHC). For LV1, brain scores with positive saliences indicate a predominant correspondence of a region’s connectivity to the anterior network, whereas negative brain scores indicate a predominant correspondence to the posterior network. For LV2, brain scores with positive saliences indicate a predominant correspondence of a region’s connectivity to the network shared by the aHC and pHC, whereas negative brain scores indicate a predominant correspondence to the network primarily linked to themHC.

Brain scores for the aHC, mHC, and pHC from LV1 and LV2 were used as predictors of episodic memory in a multivariate analysis of variance (ANOVA) including performance on the 3 episodic subtests, as well as the composite episodic memory score, as dependent variables (controlling for sex, mean FD, and SNR; reported *P-*values are unadjusted and results should be considered statistically significant at a Bonferroni-corrected level of *P* < 0.01). None of the episodic memory measures were successfully predicted by the model of participants’ network expression (episodic composite: *F*_11,165_ = 1.56, *P* = 0.115; word recall: *F*_11,165_ = 1.40, *P* = 0.176; number–word recall: *F*_11,165_ = 1.72, *P* = 0.074; object–location recall: *F*_11,165_ = 1.26, *P* = 0.256). At zero order, LV1 aHC brain scores showed negative correlations with both composite episodic memory (*r* = −0.15, *P* = 0.048) and number–word recall (*r* = −0.18, *p* = 0.022). Such negative zero-order correlations were similarly observed for LV1 mHC brain scores (episodic composite: *r* = −0.18, *P* = 0.017; number–word recall: *r* = −0.22, *P* = 0.004), indicating that the greater the negative aHC and mHC brain scores—conveying a coherence of connectivity to the posterior network rather than the anterior network—the greater the memory performance. Only the correlation between mHC brain scores and number-word recall was significant at the Bonferroni-corrected level of *P* < 0.01 and remained significant after controlling for mHC GM volume and perfusion (*r* = −0.25, *P* = 0.001).

#### Axis-Dependent Connectivity and Hippocampal Dopamine D2DR

Hippocampal D2DR was used as a predictor of aHC, mHC, and pHC network expression in a multivariate ANOVA including brain scores from LV1 and LV2 as dependent variables (controlled for sex, mean FD, and SNR). The overall effect of hippocampal D2DR on network expression was not significant (*F*_6,167_ = 1.53, *P* = 0.173), although D2DR showed a tendency for being negatively related to both aHC (*F*_1,167_ = 2.88, *P* = 0.092, β = −43.34) and mHC (*F*_1,167_ = 3.68, *P* = 0.057, β = −51.93) brain scores from LV1, suggesting that higher levels of D2DR were linked to lower adherence of aHC and mHC connectivity to the anterior network.

### Memory- and D2DR-Related Hippocampal Connectivity

Apart from the negative correlation between mHC network expression and performance on the number–word recall test, the anterior and posterior networks identified by LV1 were overall unrelated to both episodic memory performance and hippocampal D2DR. Given the interrelatedness of MTL functional connectivity, episodic memory, and hippocampal D2DR previously reported by Nyberg et al. (2016), it is, however, likely that the hippocampus indeed shows large-scale connectivity associated with both memory performance and D2DR. In order to concomitantly examine axis-related connectivity, episodic memory, and hippocampal D2DR we ran a second, behavioral PLS analysis including episodic memory performance and hippocampal D2DR as covariates together with the correlation maps belonging to the aHC, mHC, and pHC. Whereas the first PLS sought to identify differences in connectivity across the aHC, mHC, and pHC, the second PLS sought to identify how connectivity of each of these regions was related to episodic memory and D2DR. Based on the findings from Nyberg et al. (2016), demonstrating that caudate-MTL connectivity mediating the association between episodic memory and D2DR was localized in anterior MTL regions, we hypothesized that the hippocampus would show connectivity linked to both memory performance and D2DR that this would extend to include striatal regions and that it would potentially be most pronounced in connectivity of theaHC.

**Figure 3:**
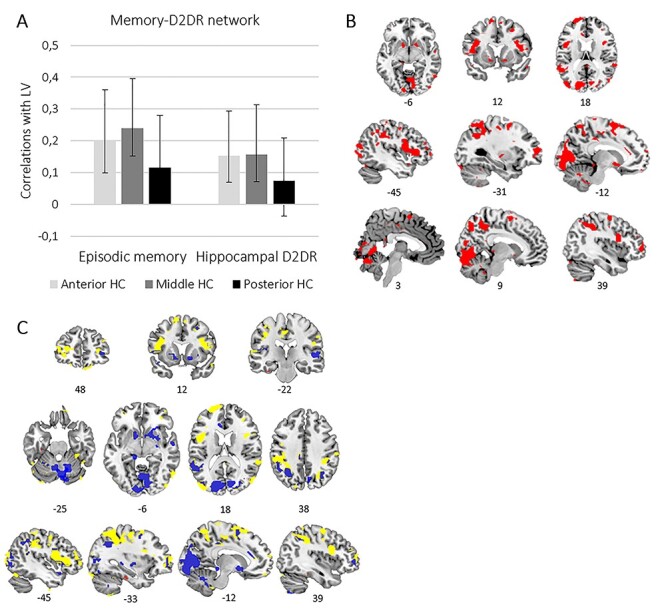
A network associated with episodic memory and hippocampal D2DR across hippocampal regions. (*A*) For each region, bars (with 95% CI) represent the correlation of episodic memory and hippocampal D2DR with the pattern exhibited by the LV. (*B*) Brain regions (in red) displaying memory- and D2DR-related connectivity with the 3 hippocampal regions. (*C*) The memory–D2DR network in relation to the anterior and posterior networks from LV1 of the first PLS. Regions of the memory–D2DR network overlapping with the anterior network (red), with the posterior network (blue), and regions specific to the memory–D2DR network (yellow).

The behavioral PLS analysis yielded 1 significant LV (explaining 68% of the variance, *P* < 0.001) exhibiting a network positively associated with both episodic memory and hippocampal D2DR. This network was equally expressed in connectivity of the 3 hippocampal regions, even though the association of hippocampal D2DR to this network just fell short of significance for the pHC (CIs crosses zero; Fig. 3). This memory–D2DR network extended to widespread areas including the lingual gyrus, precuneus, PCC, inferior and superior frontal gyrus, middle temporal gyrus, middle occipital gyrus, fusiform gyrus, and inferior parietal gyrus. This network also included the retrosplenial cortex, angular gyrus, anterior cingulate cortex (ACC), the putamen, and caudate nucleus (Table 3).

**Table 3:** Regions in the memory- and DA-related hippocampal network

	x, y, z	cluster size	BSR
Lingual gyrus, R	6, −66, −3	4565	7.3
Lingual gyrus, L	−6, −72, 6		5.7
Precuneus, R	12, −48, 45		7.0
Precuneus, L	−9, −60, 63		4.7
Calcarine fissure, L	−9, −84, 3		5.8
Superior parietal gyrus, L	−33, −54, 57		5.6
Superior parietal gyrus, R	21, −66, 63		5.7
Supplemental motor area, L	−15, 6, 63		5.7
Superior occipital gyrus, R	24, −87, 33		5.6
Cerebellum, L	−12, −48, −39		5.5
Supramarginal gyrus, L	−48, −36, 33		5.2
Angular gyrus, L	−27, −54, 36		5.2
Middle temporal gyrus, L	−66, −33, 6		5.1
Middle occipital gyrus, L	−39, −87, 15		5.1
Middle frontal gyrus, L	−33, 6, 60		4.9
Superior temporal gyrus, L	−60, −45, 15		4.9
Caudate nucleus, L	−12, 15, −9		4.6
Caudate nucleus, R	9, 9, −6		3.8
Postcentral gyrus, L	−42, −30, 60		4.6
Cuneus, L	−12, −84, 15		4.6
Precentral gyrus, L	−30, −15, 51		4.5
Inferior parietal gyrus, L	−33, −42, 45		4.4
Inferior parietal gyrus, R	39, −51, 39		4.0
Fusiform gyrus, L	−36, −78, −15		4.2
Middle cingulate cortex, L	−3, −18, 42		4.1
Middle cingulate cortex, R	9, −27, 45		3.7
PCC, R	1, −42, 18		3.5
BA30/retrosplenial cortex, L	−3, −45, 21		3.0
Inferior frontal gyrus, L	−45, 9, 9	379	5.2
Insula, L	−39, −3, 9		4.6
Putamen, L	−30, −9, 3		4.1
Superior frontal gyrus, L	−27, 60, 15	341	5.0
Middle orbitofrontal gyrus, L	−33, 48, −3		4.6
Anterior cingulate cortex	0, 42, 18		3.5
Precentral gyrus, R	33, −6, 57	176	5.6
Middle frontal gyrus, R	30, 12, 48		4.5
Middle temporal gyrus, R	57, −60, 0	148	5.4
Inferior frontal gyrus, R	45, 12, 12	120	4.7
Middle frontal gyrus, R	33, 51, 3	114	5.4
Putamen, R	30, 3, −6	89	4.0
Supplemental motor area, R	6, 3, 63	71	5.2
Superior temporal gyrus, R	57, −39, 12	58	5.3
Middle occipital gyrus, R	48, −78, 12	54	4.3
Pallidum, L	−12, 6, −6	51	5.3
Rectus, L	−6, 57, −18	41	4.6
Thalamus, R	12, −30, 3	34	5.0
Middle orbitofrontal gyrus, R	18, 51, −18	33	4.7
Parahippocampal gyrus, L	−15, −30, −9	33	4.9
Superior temporal gyrus, R	57, −6, −9	27	4.0
Paracentral lobule, L	−9, −30, 78	24	4.6
Middle temporal gyrus, L	−54, −60, 3	22	4.1
Fusiform gyrus, R	33, −36, −24	21	4.9
Superior temporal pole, L	−45, 12, −15	12	4.0
Middle temporal pole, R	57, 9, −21	12	4.3
Inferior parietal gyrus, R	60, −48, 42	10	3.8

Note: Regions presented indented and without specified cluster size are subsidiary maxima of main clusters. Minimum cluster size = 10 voxels.

The pattern of correlations observed for brain scores with episodic memory and D2DR across hippocampal regions remained the same after regressing SNR out of the brain scores (*r*s for episodic memory: aHC: 0.186 vs. 0.192; mHC: 0.228 vs. 0.226; pHC: 0.117 vs. 0.101; D2DR: aHC: 0.128 vs. 0.148; mHC: 0.138 vs. 0.151; pHC 0.039 vs. 0.061).

### Integrating Individual Differences in Axis-Specific and Axis-Common Hippocampal Connectivity

Since our 2 PLS analyses were used to identify different types of hippocampal connectivity—the first identifying connectivity primarily differing across anterior–posterior regions (which showed limited associations with memory and D2DR); the other identifying connectivity primarily explaining individual differences in episodic memory and D2DR (which was equally expressed by anterior–posterior regions)—we next addressed the question to what extent the networks observed in the 2 analyses were concomitantly expressed at an individual level. More specifically, we explored whether axis-specific connectivity (conveyed by LV1 of the first PLS) varied between individuals in a manner relevant to participants’ expression of connectivity associated with memory and D2DR (conveyed by the secondPLS).

**Figure 4:**
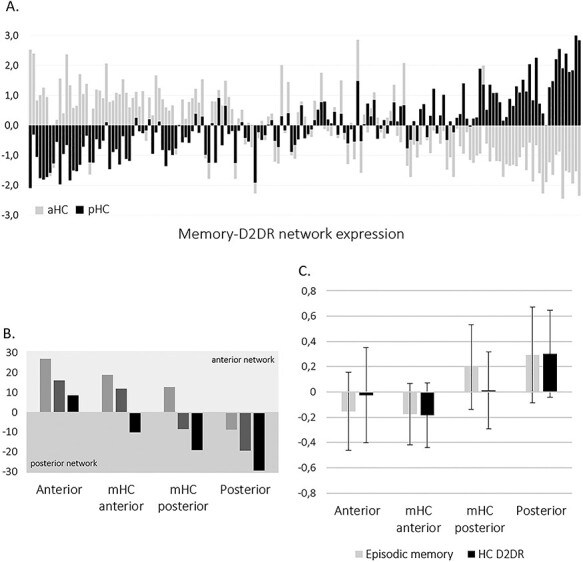
(*A*) Brain scores of the anterior and posterior hippocampus from the first PLS analysis (controlled for sex, mean FD, and SNR; *z*-standardized; pHC brain scores were reversed as to simply convey magnitude) plotted across participants ranked by expression of the memory–D2DR network. (*B*) Brain scores from LV1 across 4 groups displaying different combinations of anterior, middle, and posterior hippocampal connectivity. (*C*) Mean episodic memory performance and hippocampal D2DR (with 95% CIs) across connectivity groups.

Taking a descriptive approach, we inspected LV1 brain scores pertaining to aHC and pHC connectivity as distributed across individuals’ expression of the memory–D2DR network. Since the aHC, mHC, and pHC were similarly associated with the memory–D2DR network, an average of the 3 regions’ brain scores was calculated. The variables sex, mean FD and SNR were regressed out of this average brain score measure. Figure 4*A* shows z-standardized brain scores (after controlling for sex, mean FD and SNR) of the aHC and pHC from LV1 of the first PLS plotted across participants ranked according to their expression of the memory–D2DR network. Notably, high expression of the memory–D2DR network was paralleled by low adherence of aHC connectivity to the anterior network, in combination with high adherence of pHC connectivity to the posterior network (Fig. 4*A*).

Following this observation, indicating marked individual differences in participants’ combined aHC and pHC connectivity, we categorized participants into groups based on the combination of their aHC, mHC, and pHC brain scores from LV1 of the first PLS (Fig. 4*B*). This resulted in 2 groups displaying connectivity adhering to the anterior–posterior division conveyed by LV1: one with mHC connectivity corresponding to the anterior network (n = 55) and one with mHC connectivity corresponding to the posterior network (n = 41), as well as 2 groups showing similar connectivity across hippocampal regions: one for which connectivity of all regions corresponded to the anterior network (n = 35), and one for which connectivity of all regions corresponded to the posterior network (n = 36). Three individuals displayed mHC connectivity opposite to connectivity of the aHC and pHC, and were not included in the four described groups.

Highest levels of episodic memory and hippocampal D2DR were observed in participants displaying shared connectivity with the posterior network across hippocampal regions (Fig. 4*C*). Episodic memory was significantly lower in participants displaying shared connectivity with the anterior network across hippocampal regions (*t* = 1.86, *p* = 0.034, 1-tailed). In line with the observed negative correlation between mHC connectivity and episodic memory performance, comparing the 2 groups expressing the anterior–posterior division in connectivity, but differing in terms of mHC connectivity, showed that expressing mHC connectivity adhering to the posterior network rather than the anterior network was linked to superior memory performance (*t* = 1.86, *p* = 0.033, 1-tailed).

### Hippocampal Networks in Relation to Volume and Perfusion

Volume significantly differed between hippocampal regions, such that aHC < mHC (*t* = −97.90, *P* < 0.001) and mHC > pHC (*t* = 51.16, *P* < 0.001), reflecting the different sizes of aHC, mHC, and pHC ROIs. The mHC displayed greatest mean volume (2178 ± 257 mm^3^), followed by the pHC (1228 ± 146 mm^3^), and lastly the aHC (522 ± 79 mm^3^). Volume of these regions was used as predictors of network expression in a multivariate ANOVA including brain scores from LV1 and LV2 of the first PLS as dependent variables (controlled for sex, mean FD, and SNR). None of the regions’ volume successfully predicted network expression (aHC GM: *F*_6,165_ = 0.89, *P* = 0.501; mHC GM: *F*_6,165_ = 0.61, *P* = 0.725; pHC GM: *F*_6,165_ = 0.13, *P* = 0.993), with no significant zero-order associations between volume and brain scores. A second model included brain scores from the memory–D2DR network as dependent variables, but similarly showed no effect of GM on network expression (aHC GM: *F*_6,165_ = 0.56, *P* = 0.644; mHC GM: *F*_6,165_ = 0.29, *P* = 0.831; pHC GM: *F*_6,165_ = 0.10, *P* = 0.958).

Similarly, perfusion was the highest in the mHC (*M* = 42.91) and significantly lower in both the aHC (*M* = 40.31; *t* = −8.43, *P* < 0.001) and pHC (*M* = 41.84; *t* = −3.31, *P* < 0.001). Perfusion of the 3 hippocampal regions showed no effects on brain scores from either the first PLS (aHC perfusion: *F*_6,167_ = 0.82, *P* = 0.555; mHC perfusion: *F*_6,167_ = 0.78, *P* = 0.589; pHC perfusion: *F*_6,167_ = 0.55, *P* = 0.770), nor the second (aHC perfusion: *F*_6,167_ = 0.66, *P* = 0.651; mHC perfusion: *F*_6,167_ = 0.76, *P* = 0.584; pHC perfusion: *F*_6,167_ = 0.47, *P* = 0.801).

## Discussion

The primary aim of this study was to characterize axis-dependent large-scale hippocampal networks and their relevance to episodic memory in older adults. Moreover, we assessed to what extent DA, as conveyed by hippocampal D2DR, modulated such networks and their association with memory performance. Using PLS we identified 2 dissociated resting-state networks differentially linked to connectivity of the anterior and posterior hippocampus. These were organized in correspondence with axis-specific networks established in younger adults (Kahn et al. 2008; Poppenk and Moscovitch 2011; Libby et al. 2012; Grady 2019) but showed only limited associations with memory and D2DR. Instead, a memory-related and D2DR-modulated network was found in connectivity common to regions along the anterior–posterioraxis.

In line with previous findings of an anterior–temporal/posterior–medial division of hippocampal connectivity in older adults (Das et al. 2015), we observed dissociated anterior and posterior networks connected to regions along the hippocampal axis in a gradient-like manner. Connectivity of the anterior hippocampus primarily contributed to a network including anterior temporal and orbitofrontal regions, whereas connectivity of the posterior hippocampus contributed to a more extensive network including parietal, occipital, and medial frontal areas. The nonsignificant contribution of middle hippocampal connectivity to the dissociation between networks indicates that both anterior and posterior networks might be equally represented in connectivity of this region. This is consistent with the middle hippocampus possibly functioning as a transition area, where connectivity with anterior and posterior networks is integrated by way of the region’s common connectivity with the perirhinal and parahippocampal cortex (Maass et al. 2015; Zhuo et al. 2016). In support of such integration promoting episodic memory function, connectivity of the middle hippocampus did indeed show a significant association with memory performance.

The spatial similarity of the identified anterior and posterior networks to connectivity demonstrated in young adults (Kahn et al. 2008; Poppenk and Moscovitch 2011; Libby et al. 2012; Persson et al. 2018; Grady 2019) indicates that the general organization of axis-dependent networks might be preserved in older age (at least up to 68 years). Moreover, there were no associations between networks and either hippocampal volume or perfusion. However, even though we found no evidence for individual differences in network expression being accounted for by hippocampal volume or perfusion, this does not rule out the possibility of hippocampal connectivity being associated with volume and perfusion, when conveyed in a different manner than by our current PLS models. Given that our sample consists of relatively young older adults, it is possible that age-related alterations in hippocampal networks might occur at a later stage in life, at a point of more advanced hippocampal atrophy and decline of the D2DR system. For instance, hippocampal atrophy has been reported to induce reorganization of memory-related cortical activation, only after reaching a critical threshold (Pudas et al. 2018). To what extent organization of anterior and posterior networks alters in aging remains to be addressed by studies investigating age-related changes across a wider age span and several time points.

Contrary to our predictions, we found limited evidence for hippocampal D2DR modulating hippocampal regions’ connectivity with anterior and posterior networks, which would have been consistent with the enhancing role of DA on distinctiveness of functional networks (Seamans and Yang 2004; Shafiei et al. 2019; Shine et al. 2019). Based on findings linking caudate–hippocampus connectivity, D2DR, and episodic memory together (Nyberg et al. 2016), we nevertheless hypothesized that the hippocampus would show large-scale memory-related connectivity modulated by D2DR. A behavioral PLS indeed identified a widespread network significantly associated with both episodic memory performance and D2DR. This network was similarly evident in connectivity of the anterior, middle, and posterior hippocampus, and in accordance with our predictions, included the striatum. A role of hippocampal–striatal connectivity in episodic memory is consistent with findings of hippocampal–caudate interactions during episodic memory encoding (Sadeh et al. 2010), and similar contributions of these regions to subsequent memory effects (Ben-Yakov and Dudai 2011). Novelty- and motivation-dependent dopaminergic release by midbrain regions, affecting long-term potentiation in the hippocampus, likely constitutes a possible mechanism behind these observations (Lisman and Grace 2005; Adcock et al. 2006; Shohamy and Adcock 2010). Frank et al. (2019) recently provided indications thereof by demonstrating that MTL–ventral striatum connectivity predicted individual differences in reward modulation of memory. Given that connectivity of the caudate nucleus mediating the association between episodic memory and D2DR was previously found in anterior regions of the MTL (Nyberg et al. 2016), we anticipated memory- and D2DR-related connectivity to possibly vary in an axis-dependent manner. However, the results indicated that D2DR equally modulates memory-related hippocampal connectivity across anterior–posterior regions.

In addition to the striatum, the memory–D2DR network extended to widespread cortical areas, including posterior regions of the DMN, such as the PCC, inferior parietal cortex, precuneus, and angular gyrus, as well as the retrosplenial cortex reported to function as a mediator of DMN–MTL connectivity (Kaboodvand et al. 2018). Medial prefrontal connectivity was evident in the ACC, rectus, and the superior frontal gyrus. Consequently, this network overlapped with the posterior–medial network considered predominantly linked to the posterior hippocampus (Ranganath and Ritchey 2012; Ritchey and Cooper 2020), corresponding well to a core network implicated in various functions central to episodic and autobiographical memory (Svoboda et al. 2006; Schacter et al. 2007; Spreng et al. 2008; Benoit and Schacter 2015).

Critically, such a network was reflected also in the posterior network identified by the initial PLS—although hippocampal regions’ connectivity with this network showed only minor associations with episodic memory. A stronger link between superior memory performance and high expression of the posterior network—consistent with memory performance being recollection based, likely benefitting from the detail-oriented processing attributed to regions of the posterior–medial network (Poppenk and Moscovitch 2011; Ranganath and Ritchey 2012; Ritchey and Cooper 2020)—was, however, established through concomitant assessment of brain scores from the first and the second PLS. Demonstrating commonalities across results, individuals showing high expression of the memory–D2DR-related network also displayed both anterior and posterior hippocampal connectivity predominantly adhering to the posterior network. Assessment of individual differences in the combination of anterior, middle, and posterior connectivity from the initial PLS yielded complementary results. Levels of episodic memory performance and D2DR were greatest in those showing shared connectivity with the posterior network across all 3 hippocampal regions. Shared connectivity with the anterior network across hippocampal regions was in contrast linked to significantly inferior memory performance.

While we remain cautious in drawing conclusions based on our descriptive observations, we speculate that age-related deterioration of anterior and posterior network integrity (Das et al. 2015), as well as axis-related variation in age effects on hippocampal structure and function (Ta et al. 2012; Malykhin et al. 2017; Nordin et al. 2017), constitute possible sources of individual differences in adherence of anterior, middle, and posterior hippocampal connectivity to anterior and posterior networks. For instance, previous studies report predominant age-related cortical decoupling of the posterior hippocampus (Damoiseaux et al. 2016), negatively impacting episodic memory (Salami et al. 2016), as well as elevated anterior hippocampus–prefrontal connectivity during encoding in older individuals at risk of pathological aging (Nyberg et al. 2019).

A potential limitation of our connectivity assessments was that the number of voxels constituting hippocampal ROIs significantly differed between anterior, middle, and posterior regions. Since the signal from the anterior hippocampus was based on a lower number of voxels than for the middle and posterior hippocampus, this might suggest that correlation maps were more reliably estimated for middle and posterior, compared with anterior, hippocampal regions. However, controlling analyses for estimates of each region’s SNR did not change our results, suggesting that a lower number of voxels may not radically impact the patterns observed byPLS.

In conclusion, our results provide evidence for axis-dependent organization of hippocampal connectivity in older age and support a role of hippocampal connectivity with a posterior–medial network in episodic memory function. Importantly, hippocampal connectivity accounting for individual differences in episodic memory was not primarily axis specific, but rather characterized by connectivity engaging the full extent of the hippocampal axis. Moreover, memory-related hippocampal connectivity modulated by D2DR included posterior parietal, prefrontal, and striatal regions, extending previous findings by bringing observations of memory-related hippocampal–striatal connectivity into the context of large-scale network organization.

## Funding

Swedish Research Council, Umeå University; Umeå University–Karolinska Institute Strategic Neuroscience Program; Knut and Alice Wallenberg Foundation; Torsten and Ragnar Söderberg Foundation; Alexander von Humboldt Research award; Jochnick Foundation; Swedish Brain Power; Swedish Brain Foundation; Västerbotten County Council; Innovation Fund of the Max Planck Society; and the 2010 Gottfried Wilhelm Leibniz Research Award from the German Research Foundation (DFG).

## Notes

*Conflict of Interest*: None declared.

## Supplementary Material

Supplementary_materials_Nordin_etal_bhab023
