# Supplementary material for: Distinct and Common Large-Scale Networks of the Hippocampal Long Axis in Older Age: Links to Episodic Memory and Dopamine D2 Receptor Availability
Source: Cereb Cortex. 2021 Mar 2;31(7):3435–50. doi: 10.1093/cercor/bhab023 (PMC8196260; doi:10.1093/cercor/bhab023)
Supplement: Supplementary_materials_Nordin_etal_bhab023 [file supplementary_materials_nordin_etal_bhab023.pdf]

Visualizing whole-brain connectivity for each hippocampal region

A mean image of participants’ correlation maps were created and thresholded at 0.20, for each hippocampal region, respectively. Resulting patterns of whole-brain connectivity for each hippocampal region are visualized in Supplementary Figure 1.

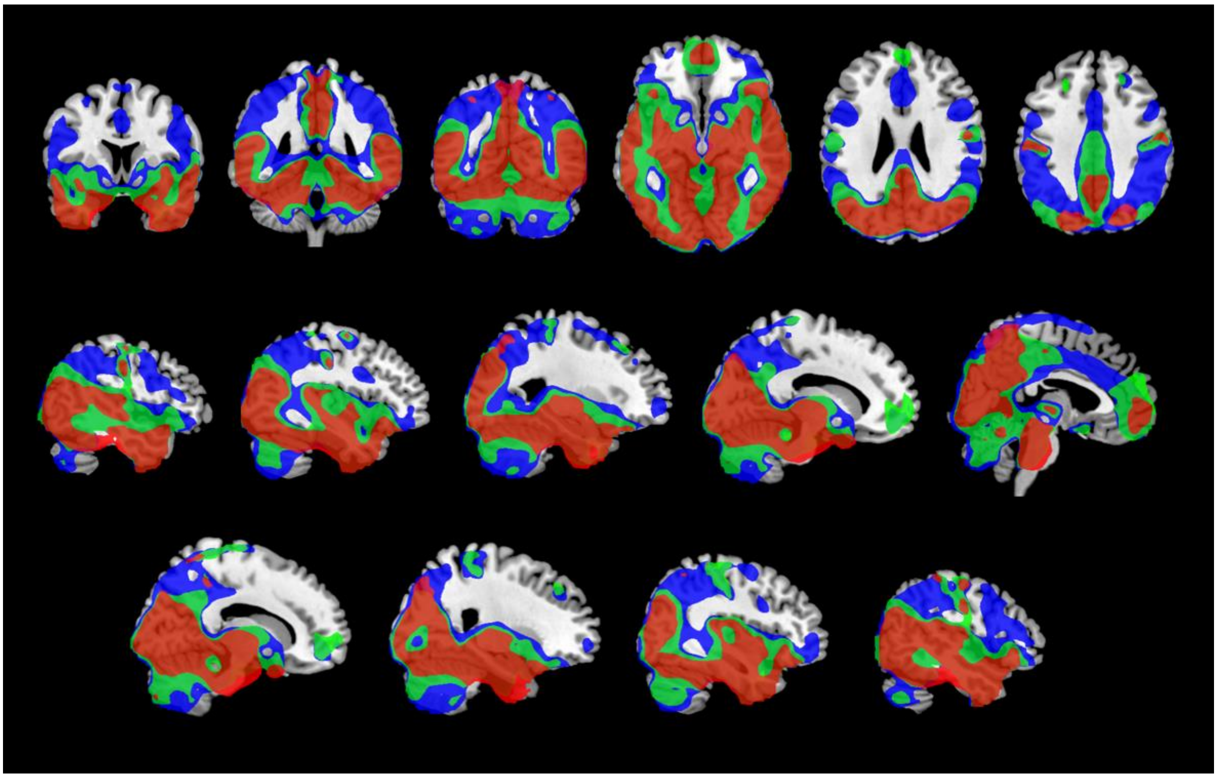

Supplementary Figure 1. Overlaid mean functional connectivity maps for the anterior (red), middle (green), and posterior (blue) hippocampus.

PLS analyses using non-smoothed resting-state data

A PLS analysis using non-smoothed data assessed connectivity across anterior, middle, and posterior hippocampal regions. A primary LV explaining 77.2% of the variance ($p < .001$) was identified, together with a second significant LV explaining 22.8% of the variance ($p = .001$). The first LV conveyed a pattern dissociating anterior and posterior networks, while the second LV conveyed a pattern dissociating a network primarily linked to the mHC, from a network primarily linked to the aHC and pHC. While results corresponded well to the results presented in the main manuscript, conveying a gradient-like organization of hippocampal connectivity with anterior and posterior networks, one observed difference was that connectivity of the mHC in this version significantly contributed to the anterior network.

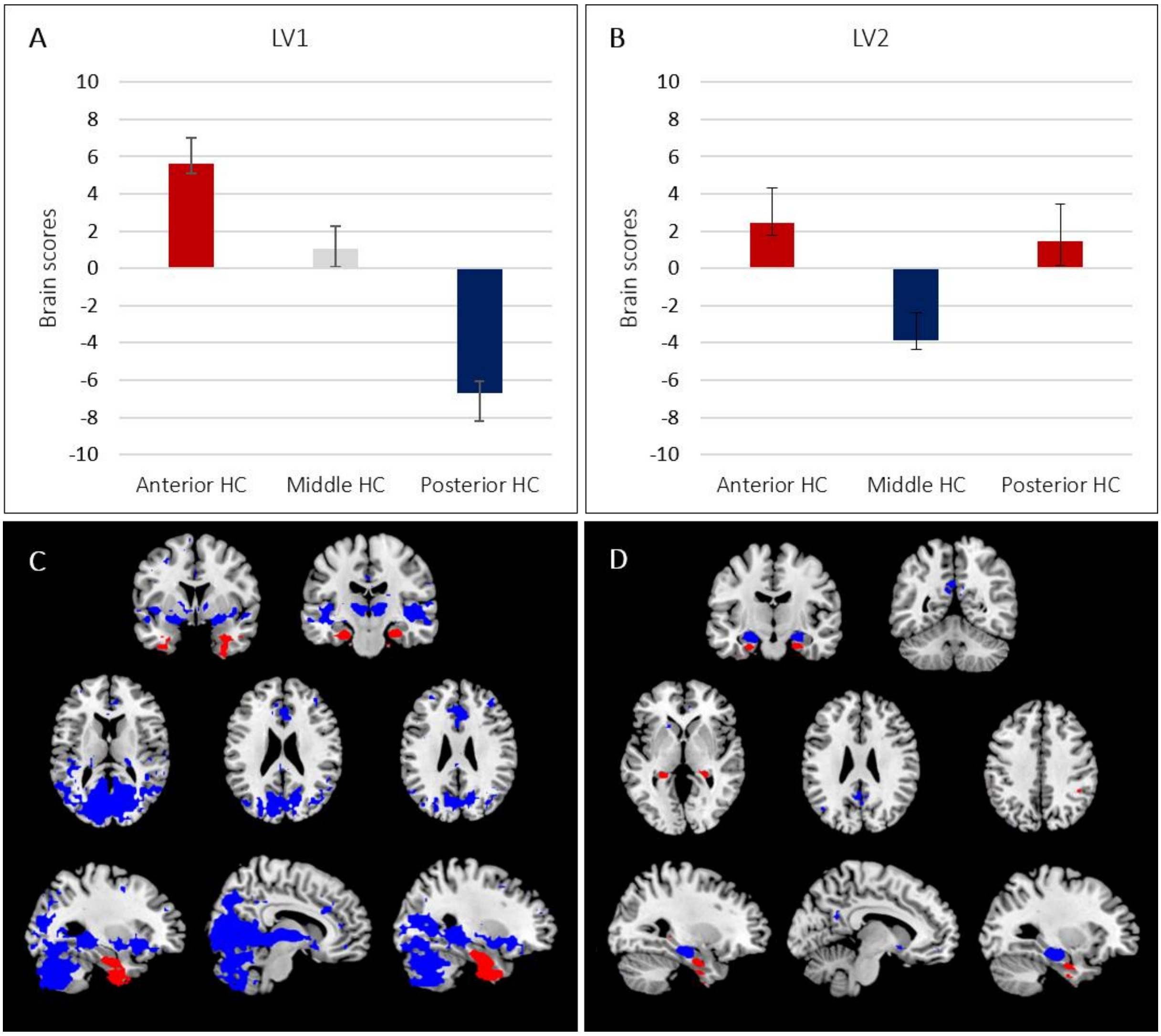

Supplementary Figure 2. A) Dissociation of anterior and posterior hippocampal connectivity. Bars (with 95% CI) represent mean brain scores pertaining to connectivity of each hippocampal region, expressing associations between connectivity of each region and the pattern exhibited by LV1. B) Dissociation of middle hippocampal connectivity from connectivity shared by the anterior and posterior hippocampus. Bars (with 95% CI) represent mean brain scores pertaining to connectivity of each hippocampal region, expressing associations between each regions' connectivity and the pattern exhibited by LV2. C) Spatial pattern corresponding to LV1: regions primarily connected to the anterior hippocampus (red); to the posterior hippocampus (blue). D) Spatial pattern corresponding to LV2: connectivity shared by the anterior and posterior hippocampus (red); regions primarily connected to the middle hippocampus (blue).

A second, behavioral PLS, assessed connectivity linked to episodic memory and hippocampal D2DR, and identified a significant LV explaining 49.1% of the variance (p < .001). This LV expressed a network linked to both episodic memory and D2DR, similarly evident in connectivity of the anterior, middle, and posterior hippocampus.

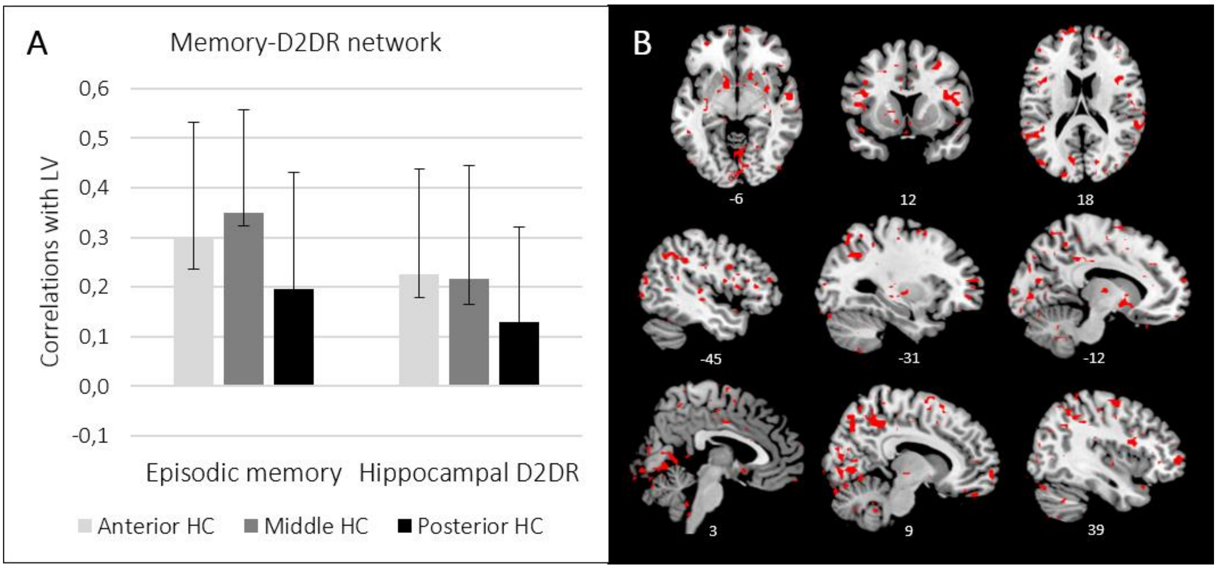

Supplementary Figure 3. A network associated with episodic memory and hippocampal D2DR across hippocampal regions. A) For each region, bars (with 95% CI) represent the correlation of episodic memory and hippocampal D2DR with the pattern exhibited by the LV. B) Brain regions (in red) displaying memory- and D2DR-related connectivity with the three hippocampal regions.
